# From child social impairment to parenting stress in mothers of children with ASD: The role of parental self-efficacy and social support

**DOI:** 10.3389/fpsyt.2022.1005748

**Published:** 2022-09-06

**Authors:** Fēi Li, Mingyu Xu, Danping Wu, Yun Tang, Lingli Zhang, Xin Liu, Li Zhou, Fei Li, Liping Jiang

**Affiliations:** ^1^Department of Nursing, Xinhua Hospital Affiliated to Shanghai Jiao Tong University School of Medicine, Shanghai, China; ^2^Department of Developmental and Behavioral Pediatric and Child Primary Care, Brain and Behavioral Research Unit of Shanghai Institute for Pediatric Research, Ministry of Education-Shanghai Key Laboratory for Children's Environmental Health, Xinhua Hospital Affiliated to Shanghai Jiao Tong University School of Medicine, Shanghai, China; ^3^Faculty of Education, Yunnan Normal University, Kunming, China; ^4^Psychology and Neuroscience of Cognition Research Unit, University of Liege, Liège, Belgium

**Keywords:** autism spectrum disorder, mothers, social impairment, parenting stress, parental self-efficacy, social support

## Abstract

**Objectives:**

Children with autism spectrum disorder (ASD) can exhibit persistent deficits in social communication, causing their mothers to experience elevated parenting stress during the childrearing process. Some internal and external psychosocial resources may mediate or moderate the mother-child relationship, though the underlying mechanisms remain unclear. This study aimed to explore the predictors of parenting stress in mothers of children with ASD and elucidate the mechanisms underlying the relationship between child social impairment and parenting stress.

**Methods:**

A cross-sectional study was conducted between October 2020 and March 2022 in Shanghai, China. Mothers of children with ASD completed a survey investigating child social impairment, parenting stress, parental self-efficacy, and social support.

**Results:**

A total of 185 mothers of children with ASD were included in the final analysis. 70.27 percent of mothers experienced a clinically significant level of parenting stress. Child social impairment (*r* = 0.46, *P* < *0.001*), parental self-efficacy (*r* = −0.58, *P* < *0.001*), and social support (*r* = −0.35, *P* < *0.001*) were significantly correlated with parenting stress. Parental self-efficacy completely mediated the relationship between child social impairment and parenting stress (*B* = 0.51, *P* < 0.001), after controlling for socioeconomic status (SES) correlated with parenting stress. There was no significant moderating effect of social support between child social impairment and parenting stress (*B* = 0.01, *P* = 0.09).

**Conclusion:**

Future early intervention programs that focused on child's social communication skills and empowered mothers with related strategies through group-based parent training programs may help reduce parenting stress.

## Introduction

Autism spectrum disorder (ASD) is a neurodevelopmental disorder that typically begins to manifest in early childhood and causes lifelong impairments (1). Globally, the number of children with ASD is estimated to be at least 78 million, which brings about a considerable care burden on families and societies (2). Deficits in social communication and interaction are the most common core symptoms for autistic children (3). These children usually have difficulties expressing basic demands, initiating social interactions, and maintaining social relationships, which make their life extremely challenging (4) and leave parents highly stressed. Though the clinical manifestations and functional levels may vary depending on the severity of symptom, children with ASD generally rely on support from their caregivers (3). Numerous studies have found that parents of children with ASD experience a higher level of parenting stress than parents of neuro-typically developing children, and those with other developmental disabilities (5, 6). Parenting stress may postpone the time of early intervention (7), weaken parental engagement in parent-child interaction (8), and affect the therapeutic effect of early intervention programs (9). It is therefore necessary to explore the internal and external influencing factors of parenting stress to inform the development of targeted stress management programs.

Previous studies have identified that many factors were involved in the formation of ASD-related parenting stress (10, 11). Influencing factors may include child's severity of symptoms, the scope of emotional and behavioral behaviors, and the extent of their adaptive skills (10, 11). Among the wide range of stressors, child social impairment has been identified to independently predict parenting stress (12) and compromise parental quality of life (13). However, the majority of previous studies have recruited gender-unbalances samples, which restricts the generalizability of conclusions (5). Our recent survey of 683 mother-father dyads in ASD families showed that the psychological distress of mothers was indeed higher than that of fathers (14). Surprisingly, social impairment in children could significantly predict maternal stress but not paternal stress (14). Influenced by social expectations and divisions in the labor market, mothers usually serve as primary caregivers for children with ASD (15), and thus appear to be particularly affected by children's autistic symptoms, which implies that the psychological status of mothers should be attached greater importance.

The Individual and Family Self-management Theory (IFSMT) may serve as a theoretical frame to explain the relationship between child's autistic symptoms and parenting stress (16). According to the IFSMT framework, some contextual factors and psychosocial processes may be related to stress formation and management for ASD families (17). Contextual factors usually refer to the risk and protective factors of parenting stress, including condition-specific, individual, and family characteristics. Process factors involve knowledge and belief, self-regulation, and social facilitation. Outcomes include both proximal and distal behavior changes (e.g., parental mental health) (16). The IFSMT holds that the interplay of context, process, and outcomes shape the health condition of children with ASD and their parents (15). For the successful stress self-management of individuals, parental self-efficacy and social support are frequently reported psychosocial resources (18). As the internal resource, parental self-efficacy (PSE) is defined as parents' perceived confidence and belief in their parenting roles (19). PSE can directly affect parental psychological wellbeing and their engagement in parent-child interactions (20). Parents with a high level of PSE may maintain a relatively stable physiological index and psychological state (21). Meanwhile, due to the high demands of childrearing, parents often seek external resources to help them counteract the effect of stressors and cope with the daily challenges. Social support consists of the external provision of comfort, caring, assistance, and esteem (22), which plays an important buffering role in stressful situations (23), whereas the lack of social support may contribute to ineffective stress coping (24, 25). Based on the IFSMT, it is reasonable to assume that parental self-efficacy and social support may play a mediating and moderating role, respectively, in the relationship between child social impairment and parenting stress in mothers of children with ASD.

Despite the previously observed relationship between child's autistic symptoms and mothers' psychological stress, the roles of some psychosocial factors (e.g., parental self-efficacy and social support) in their relationships remain to be further studied. Previous studies of children with ASD tend to involve a wide age range (e.g., 4–11 years) (12), which may mask the heterogeneity of the disorder. Furthermore, it has been reported that mothers of children under the age of 6 years experienced more challenges than those of school-aged children (26). To fill the abovementioned research gaps, this study investigated parenting stress in mothers of children with ASD between 2 and 6 years old. We also explored the mechanisms underlying the effect of child social impairment on parenting stress. It was assumed that: (1) Mothers of children with ASD may experience a high level of parenting stress, which is predicted by various contextual factors (e.g., child, parent, and ASD-specific characteristics); (2) parental self-efficacy may mediate the relationship between child social impairment and parenting stress; and (3) social support may moderate the effect of child social impairment on parenting stress. We believe that a deeper insight into the mother-child relationship will inform more targeted early intervention programs, and thus further promote the overall wellbeing of autistic children and their primary caregivers.

## Methods

### Participants

This study was conducted at the Child Healthcare Department of XH Hospital, between October 2020 and March 2022. A purposive sampling strategy was adopted to recruit eligible mothers of children with ASD. Inclusion criteria were as follows: (a) mothers had a child diagnosed with ASD according to the Diagnostic and Statistical Manual of Mental Disorders, Fifth Edition (DSM-V); (b) mothers had a diagnosed child aged 2–6 years old; (c) mothers themselves were aged between 20 and 50 years old. Mothers were excluded if they (a) were diagnosed with a mental illness that may affect their participation (e.g., depression or dyslexia); (b) were non-biological parents; (c) had more than one child diagnosed with ASD. The Childhood Autism Rating Scale (CARS) (27) and Autism Diagnosis and Observation Scale (ADOS) (28) were used to verify the ASD diagnosis. The study obtained ethical approval from the institutional review board of XH Hospital, and all participants signed written informed consents before data collection.

### Procedures

When developmental and behavioral pediatricians completed the diagnostic evaluation for children at the outpatient clinic, recruitment leaflets were distributed to potentially eligible mothers of children with ASD. A research assistant explained the study procedures to mothers interested in participating. To reduce common method bias, we combined two methods of on-site and online surveys. More specifically, questionnaires for mothers regarding demographic information, parenting stress, parental self-efficacy, and social support, were administered at the outpatient clinic shortly after the diagnostic evaluation. The child questionnaire on social impairment was allowed to be submitted within the next 5 days through the ASD Management Applet, a WeChat-based platform specially designed for the ASD Cohort in our study center. Mothers were reminded *via* text message or telephone if they did not submit child questionnaires within the required time frame. Questionnaires were examined another 2 days later, and participants with incomplete questionnaires were excluded. The child questionnaires were downloaded from the online platform, and mother questionnaires were manually input into the database, with the two sources of data being precisely matched with the child's name and date of birth. All surveys combined took ~30 min to complete. Reports of the results regarding child questionnaires could be provided if parents request.

### Measures

#### Social responsiveness scale

The SRS is a 65-item parent-reported scale that measures children's deficits of social functioning in a natural context (29). Items are scored on a 4-point scale from 0 to 3, and all items can be divided into five subscales. A higher total score represents more severe social deficits. Good reliability and validity of the SRS have been established previously (30), and internal consistency in our sample was excellent (α = 0.94).

#### Parenting stress index-short form

The PSI-SF is a self-reported scale that evaluates stress regarding parenting roles (31). The PSI-SF contains 36 items that can be divided into three subscales of parental distress, parental-child dysfunctional interaction, and difficult child. Mothers rated each item on a 5-point scale from 1 (strongly disagree) to 5 (strongly agree). The total score of PSI-SF ranges from 36 to 180, and a score above 90 indicates a clinical significance (31). The Cronbach's alpha for the PSI-SF was 0.92 in this study.

#### Parental self-efficacy of competence scale

The PSOC measures the perceived belief and confidence in parental role (32). The PSOC consists of 17 items that can be divided into efficacy and satisfaction subscales. Each item is ranked on a 6-point scale from 1 (strongly disagree) to 6 (strongly agree). The items in the satisfaction subscale are reversely rated, with a higher total score representing a greater sense of competence. The reliability and validity of the PSOC in the Chinese population have been previously validated (33). The Cronbach's alpha for the PSOC was 0.74 in this study.

#### Multidimensional perceived social support scale

The MSPSS is used to assess the perception of informal support from family, friends, and significant others (34). It includes 12 items and is ranked on a 7-point scale, from 1 (very strongly disagree) to 7 (very strongly agree). The total MSPSS score ranges from 12 to 84, and a higher score represents a higher level of social support. The reliability of the MPSSS was satisfactory in previous studies (34), and the Cronbach's alpha in the present study was 0.92.

### Statistical analyses

All data analyses were performed using the SPSS 24.0 and AMOS 24.0 software (IBM Corp; Armonk). Descriptive analyses were performed for all continuous and categorical variables. We then conducted univariate analyses to compare parenting stress in mothers of children with ASD with different demographic characteristics. The associations between all variables of interest were explored with Pearson correlation analyses. Multiple linear regression analysis (enter method) was conducted to detect predictors of parenting stress for mothers, with a *P*-value of 0.05 indicating a statistical significance. To examine the mediating role of parental self-efficacy on the relationship between child social impairment and parenting stress, we implemented the bootstrapping methods with 5,000 repetitions of samples, and estimated 95% bias-corrected confidence intervals (CIs) in AMOS software. Child social impairment was included in the mediation model as the independent variable, parenting stress as the dependent variable, parental self-efficacy as the mediator, and variables correlated with parenting stress as the covariate. Meanwhile, the regression-based approach proposed by Hayes (35) was adopted to test the moderating effect of social support. In this model, child social impairment was processed as the independent variable, parenting stress as the dependent variable, social support as the moderator, variables correlated with parenting stress as the covariate, and the product of social impairment and social support as an interaction. All these variables were mean-centered to control multicollinearity.

We also tested the potential common method bias prior to statistical analyses. Results of Harman's single-factor showed that eigenvalues for 14 factors were >1, and the first factor accounted for 21.1% of the variance (lower than 50%), indicating an acceptable common method bias (36).

## Results

### Characteristics of participants

A total of 220 mothers of children with ASD were initially invited to participate, and 200 questionnaires were returned. After screening, 15 questionnaires were identified as invalid (Five mothers have children without an ASD diagnosis, six submitted questionnaires with non-negligible missing data, and four mothers had children outside the age range). There was a valid response rate of 92.5%. The mean age for children was 4.04 years (SD = 1.18), and the majority (83.8%) were male. More than half of children come from one-child families (52.4%). Ninety-four children (50.8%) were at mild-to-moderate severity of symptoms, and the remaining 91 children (49.2%) had severe symptoms. In terms of mother characteristics, the average age for the 185 mothers was 34.02 years (SD = 4.94), and 45.9% of mothers were at a moderate level of socioeconomic status (Table 1).

**Table 1:** Demographic characteristics of participants (*N* = 185).

**Characteristics**	***N* (%)**
Child age (year), M ± SD	4.04 ± 1.18
**Child gender**
Male	155 (83.8)
Female	30 (16.2)
**An only child**
Yes	97 (52.4)
No	88 (47.6)
**Child Severity of symptoms**
Mild-to-moderate	94 (50.8)
Severe	91 (49.2)
Mother's age (year), M ± SD	34.02 ± 4.94
**Mother's SES**
Low SES	50 (27.0)
Moderate SES	85 (45.9)
High SES	50 (27.0)

SES, socioeconomic status. SES was ranked in descending order, with the lowest and highest 27% of participants being classified as low SES and high SES groups, respectively. The remaining belonged to the moderate SES group.

### Descriptive and correlational analyses

The parenting stress in mothers of children with ASD scored an average of 99.48 (SD = 19.14), which was significantly higher than the cut-off scores [*t*_(184)_ = 6.74, *P* < 0.001]. The proportion of mothers with clinical stress levels (≥90) reached 70.27%. The mean scores for all variables of interest are listed in Table 2. The correlation analyses revealed statistically significant associations between parenting stress and child social impairment (*r* = 0.46, *P* < *0.001*), parental self-efficacy (*r* = −0.58, *P* < *0.001*), and social support (*r* = −0.35, *P* < *0.001*). Social impairment was significantly and negatively correlated with parental self-efficacy (*r* = −0.30, *P* < *0.001*), but not correlated with social support (*r* = −0.13, *P* = 0.08; Table 3).

**Table 2:** Mean scores for variables of interest (*N* = 185).

**Variables**	**M ±SD**
Social impairment _ total	92.97 ± 23.76
Social awareness	11.99 ± 3.03
Social cognition	18.05 ± 4.33
Social communication	34.04 ± 9.17
Social motivation	15.05 ± 4.78
Autistic mannerisms	13.83 ± 6.65
Parenting stress _ total	99.48 ± 19.14
Parental distress	34.07 ± 9.05
Parent-child dysfunctional interaction	29.5 ± 6.4
Difficult child	35.91 ± 7.53
Parental self-efficacy _ total	60.14 ± 9.71
Satisfaction	30.07 ± 7.00
Efficacy	30.06 ± 5.22
Social support _ total	60.91 ± 12.02
Family support	20.9 ± 4.83
Friend support	19.49 ± 4.82
Other support	20.53 ± 4.39

**Table 3:** Summary of correlational coefficients (*N* = 185).

**Variables**	**1**	**2**	**3**
1. Social impairment	-		
2. Parenting stress	0.46**	-	
3. Parental self-efficacy	−0.30**	−0.58**	-
4. Social support	−0.13	−0.35**	0.16*

**P < 0.001.

*P < 0.05.

### Predictors of parenting stress in mothers of children with ASD

Univariate analysis showed that there was a significant difference in parenting stress in mothers of children with severe symptoms and those with mild-to-moderate symptoms [*t*_(184)_ = 0.75, *P* < 0.001], with the former experiencing a higher level of parenting stress. Mothers who have different levels of SES also experienced significantly different levels of parenting stress [*F*_(2, 182)_ = 7.49, *P* = 0.001], and those with a lower SES were related to higher parenting stress. No significant difference was detected in parenting stress in mothers with other demographic characteristics (all *P* > 0.05). Results of multiple linear regression analyses demonstrated that child's severity of symptoms did not significantly predict parenting stress (β = 0.07, *P* = 0.23), while SES (β = −0.13, *P* = 0.02), child social impairment (β = 0.25, *P* < 0.001), parental self-efficacy (β = −0.45, *P* < 0.001), and social support (β = −0.21, *P* < 0.001) significantly predicted parenting stress. Taken together, the model explained 49.1% of total variance [*F*_(5, 179)_ = 36.45, *P* < 0.001; Table 4].

**Table 4:** Predictors of parenting stress in mothers of children with ASD.

**Variables**	***B***	**SE**	**Beta**	***t***	***P***
Constant	156.83	10.40		15.08	<0.001
Severity of symptom	2.62	2.18	0.07	1.20	0.23
SES	−3.43	1.43	−0.13	−2.40	0.02
Social impairment	0.20	0.05	0.25	4.27	<0.001
Parental self-efficacy	−0.88	0.11	−0.45	−8.05	<0.001
Social support	−0.33	0.09	−0.21	−3.79	<0.001

B, unstandardized coefficient; SE, standard error; Beta, standardized coefficient; SES, socioeconomic status.

### The mediating effect of parental self-efficacy

**Figure 1:**
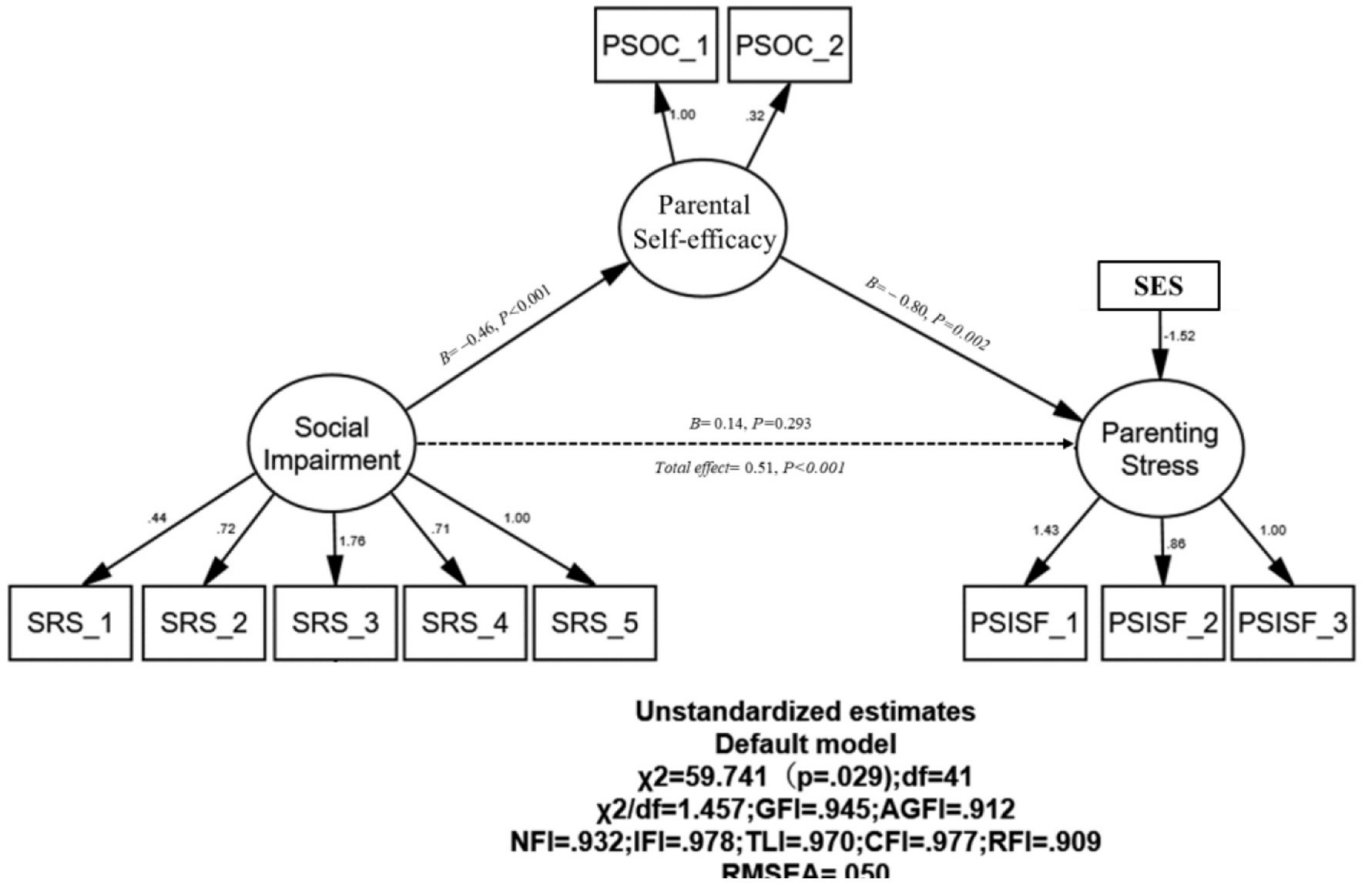
Mediation model examining the association between child social impairment and parenting stress through parental self-efficacy. SRS, The Social Responsiveness Scale; PSOC, Parental Self-efficacy of Competence Scale; PSISF, Parenting Stress Index-Short Form; SES, Socioeconomic status. The dashed line represents a non-significant effect.

The mediating role of parental self-efficacy on child social impairment and parenting stress was examined with bootstrapping analytic methods. A review of the measurement model indicated a high degree of fit to the data: $\chi_{\left(41\right)}^{2}$ = 59.74; χ^2^/*df* = 1.46, RMSEA = 0.05; SRMR = 0.06; GFI = 0.95; AGFI = 0.91; NFI = 0.93; IFI = 0.98; TLI = 0.97, CFI = 0.98, RFI = 0.91. Results demonstrated child social impairment could significantly predict parental self-efficacy (*B* = −0.46, *P* < 0.001), which further significantly and netatively predicted parenting stress (*B* = −0.80, *P* < 0.002; Figure 1). Parental self-efficacy completely mediated the relationship betweeen child social impairment and parenting stress in mothers of children with ASD (*B* = 0.51, *P* < 0.001).

### The moderating effect of social support

Results of the moderation analysis demonstrated that the interaction of child social impairment and social support did not show a significant difference (*B* = 0.01, *P* = 0.09; Table 5). Social support did not moderate the effect of child social impairment on parenting stress.

**Table 5:** Summary of moderation analysis for social support.

	**Estimate**	**SE**	***t***	***P***	**LLCI**	**ULCI**
Constant	108.12	3.50	30.89	<0.001	101.21	115.03
SES	−4.19	1.65	−2.55	0.01	−7.44	−0.94
Social impairment	0.32	0.05	6.37	<0.001	0.22	0.42
Social support	−0.42	0.10	−4.22	<0.001	−0.62	−0.23
Interaction^#^	0.01	0.004	1.70	0.09	0.00	0.02

SES, socioeconomic status.

^#^Interaction means the product of social impairment and social support.

## Discussion

This study examined how social impairment in children with ASD affected parenting stress in their mothers, under the guidance of the Individual and Family Self-management Theory (IFSMT). As we hypothesized, most mothers experienced a clinically significant level of parenting stress, which was positively correlated with child social impairment. Meanwhile, parental self-efficacy could completely mediate the relationship between child social impairment and parenting stress. Social support, however, did not significantly moderate the relationship between child social impairment and parenting stress. Some contextual factors and psychosocial variables influencing parenting stress were identified, and implications for future clinical practice were discussed.

Mothers generally act as critical informants in the early detection and diagnosis of ASD and assume primary responsibilities for childcaring (15). The core symptoms of ASD and its comorbidities leave children reliant on long-term care and supervision, making their mothers susceptible to chronic stress (37). It is, therefore, necessary to explore potential predictors of parenting stress in mothers so that targeted early intervention programs can be informed to improve their psychological functioning. This study verified that socioeconomic status (SES) could significantly predict parenting stress, and a low level of SES was linked to an elevated level of parenting stress, which corresponds to previous findings (38). SES is an indicator of family capacity to access information, support, and other services. This study defines SES as the composite index of education, occupation, family income, and place of residence. Raising a child with ASD is a demanding and resource-consuming task, as families need to advocate for additional medication, educational facilities, and early intervention services. Additional support is recommended for families in low SES, because these families have limited capacities to cope with challenges when they advocate for the affected children, and they are exposed to higher odds of mental health problems (39). Formal support in the form of government subsidies may make a difference in relieving their caregiving burden and guarantee equal access to early interventions.

Consistent with previous studies (12, 14), our results also demonstrated that child social impairment significantly predicted parenting stress in mothers. Parenting a child with ASD can be stressful because autistic children usually have difficulties expressing daily demands and suffer from behavior problems due to persistent deficits in social communication skills, which largely increases caregiving challenges for parents. The atypical parent-child interaction patterns and lack of emotional reciprocity gradually undermine maternal confidence and exacerbate parenting stress (40). It informs us that social communication skills can be the priority of early intervention programs for ASD families. In that way, we can promote the health outcomes of children and also produce a positive effect on maternal psychological wellbeing. In this study, child's severity of symptoms did not correlate with parenting stress in mothers. This seemingly contradictory phenomenon has been documented in a previous study (41), which found that parent-reported social impairment in children was significantly associated with parenting stress, but the clinician-rated severity of symptoms did not show a significant association. One possible explanation is that the severity of symptoms is a composite index that not only includes child social impairment, but also involves indicators that have not been measured in this study (e.g., cognitive ability, adaptive ability, and the emotional and behavioral problems) (41). A more comprehensive assessment of child's severity of symptoms may provide more significant implications for future clinical practice.

As primary caregivers for children with ASD, mothers' beliefs and confidence in childrearing can influence their decision-making and participation in early intervention programs. In this study, the self-efficacy in mothers of children with ASD is lower than mothers of newborns in China (42). Meanwhile, parental self-efficacy can not only significantly predict parenting stress of mothers, but also completely mediate the effect of child social impairment on parenting stress. Generally speaking, a higher level of self-efficacy is accompanied by more engagement and persistence in difficult situations (43). For families of children with ASD, parents tend to be in a state of self-doubt and confusion, as routine parenting behaviors may produce very minimal effects on their children. The cultivation of parental self-efficacy is thus an integral part of early intervention programs and may exert a positive effect on parental psychological status. Such parent support programs as Family-focused Psychoeducational Therapy (FFPT) have significantly improved parental self-efficacy and decreased psychopathological symptoms (44). During intervention programs, professionals can impart ASD-specific knowledge and social communication strategies to parents, so as to improve parents' perceived competence in the parental roles and finally alleviate parenting stress.

As an external psychosocial resource for families of children with ASD, social support may facilitate parents to coexist with their child's conditions and actively address childrearing challenges (45). Our results demonstrated that social support significantly and negatively predicted parenting stress in mothers of children with ASD. However, social support did not significantly moderate the effect of child social impairment on parenting stress, which was in contradiction to previous findings (46). Actually, the inconsistent role of social support as a moderator has recently been documented by Shepherd (47), who detected that the significance of moderation was dependent upon how social support was operationally defined. In this study, we defined social support as those from family, friends, and significant others, which was primarily provided in the form of emotional comfort instead of material support. Investigating the function of other sources of formal social support may be informative in the Chinese context, where support from official organizations was insufficient due to a high demand and limited accessibility to early intervention services.

### Limitations

This study should be interpreted with some limitations. First, as a cross-sectional study design, the sample size of the present study was relatively small, and we could not assert a causal relationship between child social impairment and parenting stress. Second, both proximal and distal outcomes should have been included according to the theoretical basis, but we did not measure the distal outcomes (e.g., anxiety and depression) of parenting stress, making studies with longitudinal design necessary. Third, this study only recruited mothers of children with ASD in an Eastern city in China, which may restrict the generalizability. Finally, all outcome measures in this study were self-reported, and influencing factors involved in this study may be limited. For future studies, objective measures of parenting stress and comprehensive inclusion of relevant factors may reach a more reliable conclusion.

## Conclusion

There is a high level of parenting stress in mothers of children with ASD, and child social impairment significantly predicts parenting stress. Parental self-efficacy and social support play a significant role in the relationship between child social impairment and parenting stress. The results inform that the domain of social communication skills for children with ASD is the priority of early intervention programs and that social skills-oriented interventions can positively affect mothers' psychological wellbeing of mothers. Parental self-efficacy may be an integral component of successful intervention programs, and the empowerment of mothers can help reduce parenting stress. Social support should be cultivated among family members and families with the same experiences, and other formal support is appealed to be in place. Based on the above findings, the present study holds that parental empowerment in group-based social skills intervention programs can reduce parenting stress in mothers of children with ASD.

## Data availability statement

The raw data supporting the conclusions of this article will be made available by the authors, without undue reservation.

## Ethics statement

The studies involving human participants were reviewed and approved by Xinhua Hospital. Written informed consent to participate in this study was provided by the participants' legal guardian/next of kin.

## Author contributions

FēL and MX proposed the research idea and study design, collected and analyzed the data, and composed the manuscript. DW reviewed and revised the draft of the manuscript. YT, LZha, and XL recruited participants, prepared data, and performed statistical analyses. LZho improved the language quality of the manuscript. FeL and LJ guided the study design and supervised the implementation process. All authors contributed to the article and approved the submitted version.

## Funding

This work was funded by the National Natural Science Foundation of China (71874107, 82125032, 81930095, and 81761128035), Shanghai Municipal Health Commission (2019SY068), the Science and Technology Commission of Shanghai Municipality (19410713500 and 2018SHZDZX01), the Shanghai Municipal Commission of Health and Family Planning (GWV-10.1-XK07, 2020CXJQ01, 2018YJRC03, and 2018BR33), and the Guangdong Key Project (2018B030335001).

## Conflict of interest

The authors declare that the research was conducted in the absence of any commercial or financial relationships that could be construed as a potential conflict of interest.

## Publisher's note

All claims expressed in this article are solely those of the authors and do not necessarily represent those of their affiliated organizations, or those of the publisher, the editors and the reviewers. Any product that may be evaluated in this article, or claim that may be made by its manufacturer, is not guaranteed or endorsed by the publisher.
